# Activation of ventral CA1 hippocampal neurons projecting to the lateral septum during feeding

**DOI:** 10.1002/hipo.23289

**Published:** 2020-12-09

**Authors:** Kenzo Kosugi, Keitaro Yoshida, Toru Suzuki, Kenta Kobayashi, Kazunari Yoshida, Masaru Mimura, Kenji F. Tanaka

**Affiliations:** ^1^ Department of Neurosurgery Keio University School of Medicine Tokyo Japan; ^2^ Department of Neuropsychiatry Keio University School of Medicine Tokyo Japan; ^3^ Section of Viral Vector Development National Institute for Physiological Sciences Okazaki Japan

**Keywords:** CA1 region, food intake, hippocampal, lateral septum nucleus, optical fibers

## Abstract

A number of studies have reported the involvement of the ventral hippocampus (vHip) and the lateral septum (LS) in negative emotional responses. Besides these well‐documented functions, they are also thought to control feeding behavior. In particular, optogenetic and pharmacogenetic interventions to LS‐projecting vHip neurons have demonstrated that the vHip^→LS^ neural circuit exerts an inhibition on feeding behavior. However, there have been no reports of vHip neuronal activity during feeding. Here, we focused on LS‐projecting vCA1 neurons (vCA1^→LS^) and monitored their activity during feeding behaviors in mice. vCA1^→LS^ neurons were retrogradely labeled with adeno‐associated virus carrying a ratiometric Ca^2+^ indicator and measured compound Ca^2+^ dynamics by fiber photometry. We first examined vCA1^→LS^ activity in random food‐exploring behavior and found that vCA1^→LS^ activation seemed to coincide with food intake; however, our ability to visually confirm this during freely moving behaviors was not sufficiently reliable. We next examined vCA1^→LS^ activity in a goal‐directed, food‐seeking lever‐press task which temporally divided the mouse state into preparatory, effort, and consummatory phases. We observed vCA1^→LS^ activation in the postprandial period during the consummatory phase. Such timing‐ and pathway‐specific activation was not observed from pan‐vCA1 neurons. In contrast, reward omission eliminated this activity, indicating that vCA1^→LS^ activation is contingent on the food reward. Sated mice pressed the lever significantly fewer times but still ate food; however, vCA1^→LS^ neurons were not activated, suggesting that vCA1^→LS^ neurons did not respond to habitual behavior. Combined, these results suggest that gastrointestinal interoception rather than food‐intake motions or external sensations are likely to coincide with vCA1^→LS^ activity. Accordingly, we propose that vCA1^→LS^ neurons discriminate between matched or unmatched predictive bodily states in which incoming food will satisfy an appetite. We also demonstrate that vCA1^→LS^ neurons are activated in aversive/anxious situations in an elevated plus maze and tail suspension test. Future behavioral tests utilizing anxious conflict and food intake may reconcile the multiple functions of vCA1^→LS^ neurons.

## 1 INTRODUCTION

The ventral hippocampus (vHip) is well known to engage in the regulation of negative emotional/affective behaviors, such as anxiety‐ (Jimenez et al., 2018), depression‐ (Bagot et al., 2015), and aversion‐related adaptive (Padilla‐Coreano et al., 2016) or defensive behaviors (Pentkowski et al., 2009). In addition to the prevailing view of vHip function, accumulating evidence indicates that the vHip also plays a role in positive emotional behaviors, as the vHip neurons have been shown to engage in food‐directed motivated behaviors (Yoshida, Drew, Mimura, & Tanaka, 2019) and appetitive behaviors (Sweeney & Yang, 2015). These multiple roles for the vHip are thought to be governed by distinct vHip subpopulations.

In terms of the output diversity from the vHip, there is a definitive vHip division between the ventral CA1 (vCA1) and vCA3 subregions. Both subregions differentially contribute to functional processes (Schumacher et al., 2018). The vCA1 has a broad range of functions related to the heterogeneity of its anatomical connections; vCA1 neurons directly project to the nucleus accumbens, medial prefrontal cortex (mPFC), hypothalamus, amygdala, and the lateral septum (LS; Bienkowski et al., 2018). Interestingly, the vCA1 communicates with other brain regions not by transmitting all information equally, but by selectively routing diverse signals according to the content and downstream targets (Ciocchi, Passecker, Malagon‐Vina, Mikus, & Klausberger, 2015).

The vHip contributes to the control of appetitive and consummatory behavior (Davidson et al., 2009), and vHip neurons form a meal memory and inhibit energy intake during the postprandial period (Hannapel, Henderson, Nalloor, Vazdarjanova, & Parent, 2017). Also, vHip neurons can induce ingestion when vHip ghrelin receptors are activated (Hsu, Suarez, & Kanoski, 2016). Several efferent pathways from the vHip, which are involved in feeding behavior, have been investigated; these include the LS, mPFC, and lateral hypothalamic area (Kanoski & Grill, 2017). In the present study, we focused on the activity of the vCA1‐LS pathway from the perspective of feeding for the following three reasons. First, pharmacological ablation of the LS significantly increases feeding behavior (Beatty & Schwartzbaum, 1968; King & Nance, 1986), indicating a role for the LS in feeding. Second, optogenetic activation of the vHip (dentate gyrus and CA3)‐LS pathway reduces food intake (Sweeney & Yang, 2015), suggesting the contribution of the vHip‐LS pathway to feeding. Third, the vCA1‐LS may link motivation and appetite since pan vCA1 neurons are associated with food‐directed motivated behaviors (Yoshida et al., 2019). Thus, the primary purpose of this study is to examine vCA1‐LS neuronal activity in feeding behavior and address the functional diversity of the vCA1.

In addition to feeding behavior, the LS plays an important role in effective coping responses to inescapable stress (Anthony et al., 2014; Singewald, Rjabokon, Singewald, & Ebner, 2011). Moreover, emerging evidence suggests potential interactions between feeding, anxiety, and stress (Maniam & Morris, 2012; Ulrich‐Lai & Ryan, 2014). Thus, the secondary aim of this study is to examine whether vCA1^→LS^ neurons are activated under stress conditions as expected, and to characterize the multiple situation‐dependent roles of vCA1^→LS^ neurons.

## 2 METHODS

### 2.1 Animals

All animal procedures were conducted in accordance with the National Institutes of Health Guide for the Care and Use of Laboratory Animals and approved by the Animal Research Committee of Keio University (approval 18082‐(0)). Experiments were carried out using 3‐ to 12‐month‐old male and female mice. All mice were maintained on a 12:12‐hr light/dark cycle (lights on at 8:00) and the behavioral experiments were conducted during the light phase. C57BL/6J mice were purchased from Oriental Yeast Co., Japan.

Htr5B‐YC double transgenic mice were obtained by crossing the *Htr5B*‐tTA (Tanaka et al., 2012) line with the tetO‐YCnano50 line (Kanemaru et al., 2014). All mouse lines are available from RIKEN BioResource Center. The genetic background of all transgenic mice was mixed C57BL/6J and 129SvEvTac. Genotyping for *Htr5B*‐tTA and tetO‐YCnano50 was previously described (Kanemaru et al., 2014; Tanaka et al., 2012).

### 2.2 Adeno‐associated virus (AAV) production and purification

AAV vectors were produced as described previously (Kobayashi et al., 2016). AAV_2_‐retro helper plasmid was kindly supplied from HHMI‐Janelia Research Campus (Ashburn; Tervo et al., 2016). The plasmid sequences are available upon request. In these experiments, AAV_2_‐retro‐hSyn‐YCnano50 (7.5 × 10^9^ vg/ml) was used to examine the vHip cell population projecting to the ventral LS by retrograde labeling.

### 2.3 Surgical procedure

Surgery was performed under anesthesia with a mixture of ketamine‐xylazine (100 mg/kg and 10 mg/kg, respectively) delivered intraperitoneally (i.p.). The anesthetized mice were fixed to a stereotaxic apparatus (Narishige). For virus infection, a glass pipette was inserted into the LS (+0.5 mm anteroposterior [AP]; +0.5 mm mediolateral [ML] from the Bregma; −2.0 mm dorsoventral [DV] from the brain surface) (Deng et al., 2019). A volume of 0.2 μl of virus solution was injected at a rate of 0.02 ml/min. After the injection was finished, the glass pipette was kept in place for 10 min. For optical recordings, mice were unilaterally implanted with an optical fiber cannula (Φ 400 μm, 0.39 NA, Thorlabs) into the vCA1 (AP –3.2 mm; ML +3.5 mm; DV –2.5 mm).

### 2.4 Lever‐press operant task

The methods for the food‐seeking lever‐press task have been described previously (Natsubori et al., 2017). Mice were housed individually under conditions of food restriction, and body weights were maintained at 85% of initial body weight. Operant training and tests were performed in an aluminum operant chamber measuring 22 cm wide, 26 cm deep and 18 cm high (Med Associates Inc.) under constant darkness. The apparatus was controlled by a computer program written in the MED‐PC language (Med Associates Inc.). A food dispenser flanked by two retractable levers was located on the floor. The lever on the left side was designated as “active” (triggering delivery of a food reward), and the one on the right was “inactive” (no relation to food reward). Each trial began with the presentation of two levers (trial start; TS). After mice pressed one active lever (LP), the levers were retracted and one food pellet was delivered (fixed ratio [FR]‐1 task). After the food delivery, a 30 s intertrial interval (ITI) was added, during which levers were retracted, followed by the automatic initiation of the next trial. Once the mice were able to obtain 50 rewards within 60 min, the training progressed to a recording session. In the recording sessions, we utilized two types of FR‐1 schedules. In Task 1, reward was given in 100% of the trials. In Task 2, reward was given in 75% of the trials. For assessment of performance under sated conditions in the FR‐1 task, mice were given free access to normal chow for 3 hr before the operant tests. Immediately after satiety, a 100% reward FR‐1 task was conducted.

### 2.5 Elevated plus maze test (EPM) test

The test apparatus consisted of two open and two closed arms (25 × 5 cm) that extended from a central platform (5 × 5 cm). Closed arms were surrounded by walls 40 cm in height. The maze was elevated 40 cm above the floor, and the room lights were turned off during testing. Mice typically avoid the open arms because they innately dislike open space (Pellow, Chopin, File, & Briley, 1985; Walf & Frye, 2007). Synchronized fiber photometry and video recordings were performed for 10 min. YC signals were compared between periods in the open arms and closed arms.

### 2.6 Tail suspension test (TST)

Mice were suspended approximately 50 cm above the floor using adhesive tape placed approximately 3 cm from the tip of their tails under constant darkness. Synchronized fiber photometry and video recordings were performed for 5 min, and behavior was manually scored as “struggling” bouts during which the mice struggled by moving their bodies.

### 2.7 Fiber photometry

The methods for fiber photometry have been described previously (Natsubori et al., 2017). An excitation light (435 nm; silver‐LED, Prizmatix) was reflected off a dichroic mirror (DM455CFP; Olympus), focused with a ×20 objective lens (NA 0.39, Olympus), and coupled to an optical fiber (M79L01, Φ 400 μm, 0.39 NA; Thorlabs) through a pinhole (Φ 400 μm). LED power was <200 μW at the fiber tip. Emitted cyan and yellow fluorescence from YCnano50 was collected via an optical fiber canula, divided by a dichroic mirror (DM515YFP; Olympus) into cyan (483/32 nm band‐pass filters, Semrock) and yellow (542/27 nm), and each was detected by a photomultiplier tube (H10722‐210, Hamamatsu Photonics). The fluorescence signals as well as the TTL signals from the behavioral set‐ups were digitized by a data acquisition module (USB‐6211, National Instruments) and simultaneously recorded using a custom LabVIEW program (National Instruments). Signals were collected at a sampling frequency of 1,000 Hz. vHip^→LS^ neuronal activity was examined in C57BL/6J mice in which vCA1 was retrogradely labeled from the LS, and pan‐vCA1 neuronal activity was analyzed using Htr5B‐YC bitransgenic mice in which YC was expressed in pan‐CA1 pyramidal neurons.

### 2.8 Data analysis and statistical analyses

Fiber photometry signals and all statistics were analyzed using MATLAB (MathWorks, MA). The fluorescence signals (yellow and cyan) were smoothed using a 100‐point moving‐average method. The YC ratio (a ratio of yellow to cyan fluorescence intensity; R) in one session was detrended using a cubic spline, and normalized within each trial whereby the Z score was calculated as (R–R_mean_)/R_SD_, where R_mean_ and R_SD_ are the mean and standard deviation of the YC ratio for each animal. Normality and equal variances were formally tested. Two‐sample comparisons were performed using a two‐sided paired *t*‐test.

### 2.9 Immunohistochemistry

Following completion of each experiment, mice were deeply anesthetized with ketamine (100 mg/kg) and xylazine (10 mg/kg) and perfused with 4% paraformaldehyde phosphate‐buffer solution. Brains were removed from the skull and post‐fixed in the same fixative overnight. Subsequently, brains were cryoprotected in 20% sucrose overnight, frozen and cut at 25 μm thickness on a cryostat. Sections were mounted on silane‐coated glass slides (Matsunami Glass, Tokyo, Japan). Sections were incubated with an anti‐green fluorescent protein (GFP) antibody (1:200, goat polyclonal; Rockland Immunochemicals Inc.) overnight at room temperature, then treated with an anti‐goat secondary antibody conjugated to Alexa Fluor 488 (1:1,000; Invitrogen) and 4,6‐diamidino‐2‐phenylindole (DAPI; 1 mg/ml, Sigma‐Aldrich Japan) for 2 hr at room temperature. Fluorescence images were obtained with an all‐in‐one microscope (BZ‐X710, Keyence).

## 3 RESULTS

### 3.1 Assessment of vHip ^→LS^ pathway‐specific activity in freely moving mice

**FIGURE 1:**
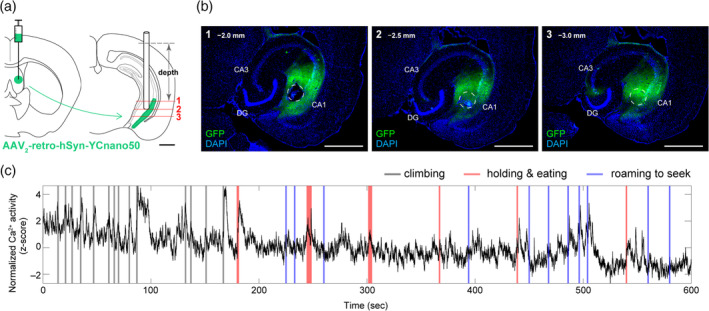
Optical measurements of neuronal activity in vHip^→LS^ input neurons under freely moving conditions. (a) Schematic illustration of the somal recordings from vCA1^→LS^ projections. AAV_2_‐retro‐hSyn‐YCnano50 virus was injected into the LS and an optical fiber was implanted into the vCA1. Scale bar, 1.0 mm. (b) The images show YCnano50 expression (green) and position of the optical fiber. YCnano50 is mainly expressed in vCA1 neurons. In this image series, the order of the horizontal sections corresponds with the numbers in the schema in (a), and the length represents the depth (mm) from the brain surface. The dashed circles indicate the location of the optical fiber. Scale bar, 1.0 mm. (c) Representative trace of compound Ca^2+^ activity in vCA1^→LS^ neurons from a freely moving mouse in its home cage. Behavioral information was manually defined based on video recordings [Color figure can be viewed at wileyonlinelibrary.com]

We first identified the vHip^→LS^ neurons by retrograde labeling. We injected AAV_2_‐retro‐hSyn‐YCnano50 into the rostral LS and examined the cell population projecting to the rostral LS (Figure 1a). Within the ventral pole of the hippocampus, CA1 neurons, but not CA3 neurons, were labeled with YC. Within the ventral CA1 neurons (vCA1), the posterior (distant from CA3) but not the anterior (close to CA3) vCA1 neurons were labeled with YC (Figure 1b).

To monitor vCA1^→LS^ neuronal activity under freely moving conditions, we implanted an optical fiber that targeted the posterior vCA1 neurons (Figure 1b) and measured compound Ca^2+^ activity using a fiber photometry technique. In the pilot study, we realized that vCA1^→LS^ neuron activation seemed to coincide with food intake. We therefore induced hunger in the mice to enhance feeding behaviors and put mice in their home cages where food pellets were placed on the floor. Mice exhibited a chain of feeding‐related behaviors consisting of roaming or climbing to seek the food, approaching the pellet, holding, and eating. vCA1^→LS^ neurons were activated during these respective behaviors; however, our visual identification of freely moving behaviors was not sufficiently reliable (Figure 1c).

### 3.2 Activity of LS projecting vCA1 neurons was increased during feeding behavior

**FIGURE 2:**
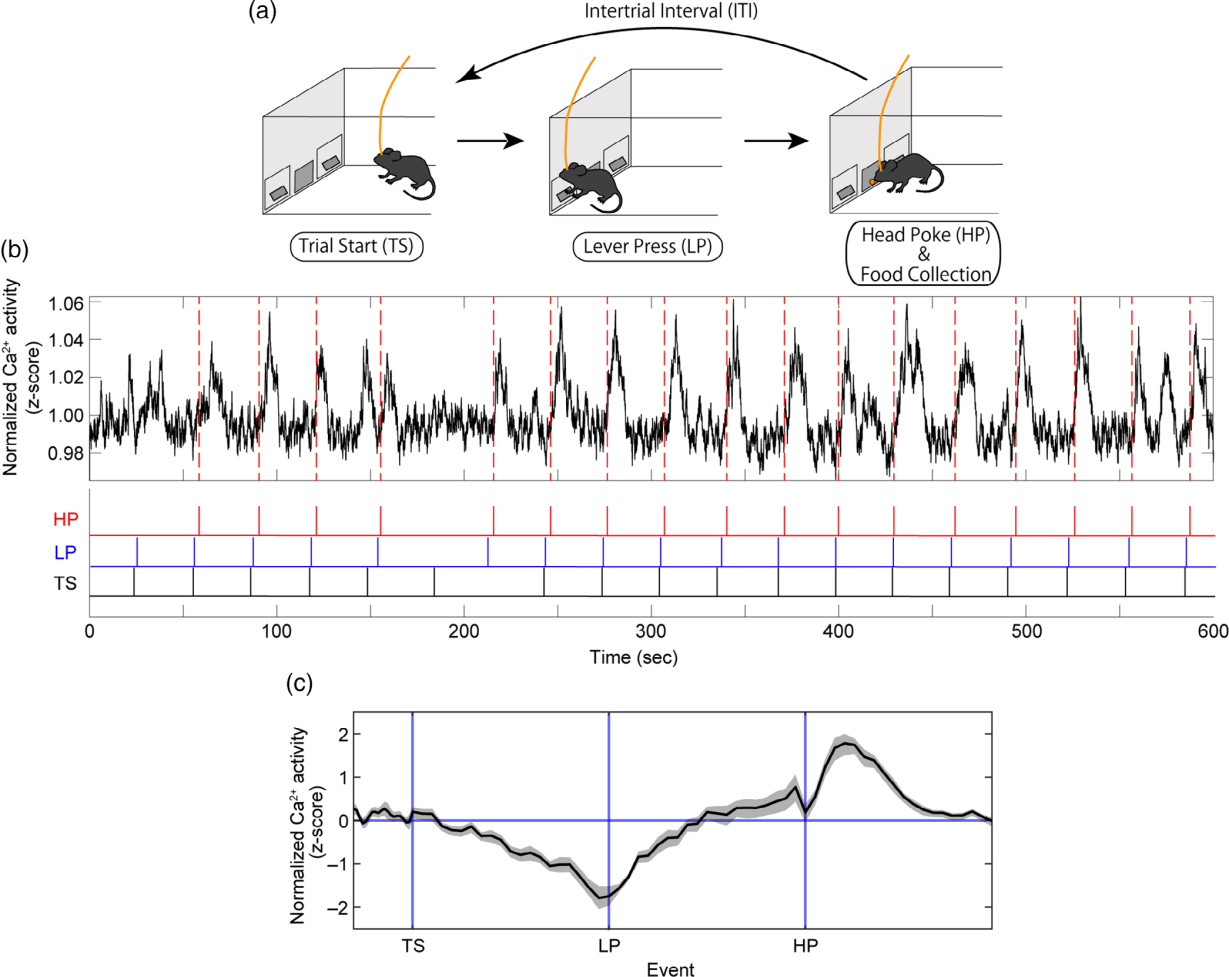
Assessment of compound Ca^2+^ activity in vCA1^→LS^ neurons during an FR‐1 operant task. (a) Schematic illustration of the FR‐1 operant task. (b) Representative Ca^2+^ activity in vCA1^→LS^ input neurons during the FR‐1 operant task (upper). The red dashed vertical lines indicate the HP timing. Vertical ticks indicate the time stamps for the TS (black), LP (blue), and HP (red) phases, respectively (lower). (c) Trace of the average Ca^2+^ signal in the vCA1^→LS^ in which the duration between trigger points was normalized (*n* = 12 mice); the shaded areas represent *SEM* [Color figure can be viewed at wileyonlinelibrary.com]

To confirm if vCA1^→LS^ neurons are activated during feeding, we employed a food‐incentive, lever‐press operant task (FR1 task) in which the preparatory, effort, and consummatory phases were clearly separated (Tanaka & Hamaguchi, 2019). In this task, mice were trained to press an active lever to obtain one palatable pellet (Figure 2a). Mice approached the lever during the preparatory phase (from the trial start time [TS] to the lever press [LP]), pressed the lever and approached the food magazine during the effort phase (from LP to the head poke [HP]), and poked their head into the magazine and ate a pellet during the consummatory phase (from HP to TS). We observed dynamic changes in event‐related compound Ca^2+^ signals from vCA1^→LS^ neurons (Figure 2b). vCA1^→LS^ activity gradually decreased during the preparatory period (TS to LP latency: median 6.2 ± 2.8 s), reached a nadir at the LP, gradually increased to the HP, and surged immediately after HP (Figure 2c). vCA1^→LS^ activity thus exhibited a peak (6.0 ± 4.2 s after HP) and returned to the baseline (23.7 ± 5.8 s after HP). These data demonstrate that vCA1^→LS^ neuronal activation coincides with feeding.

**FIGURE 3:**
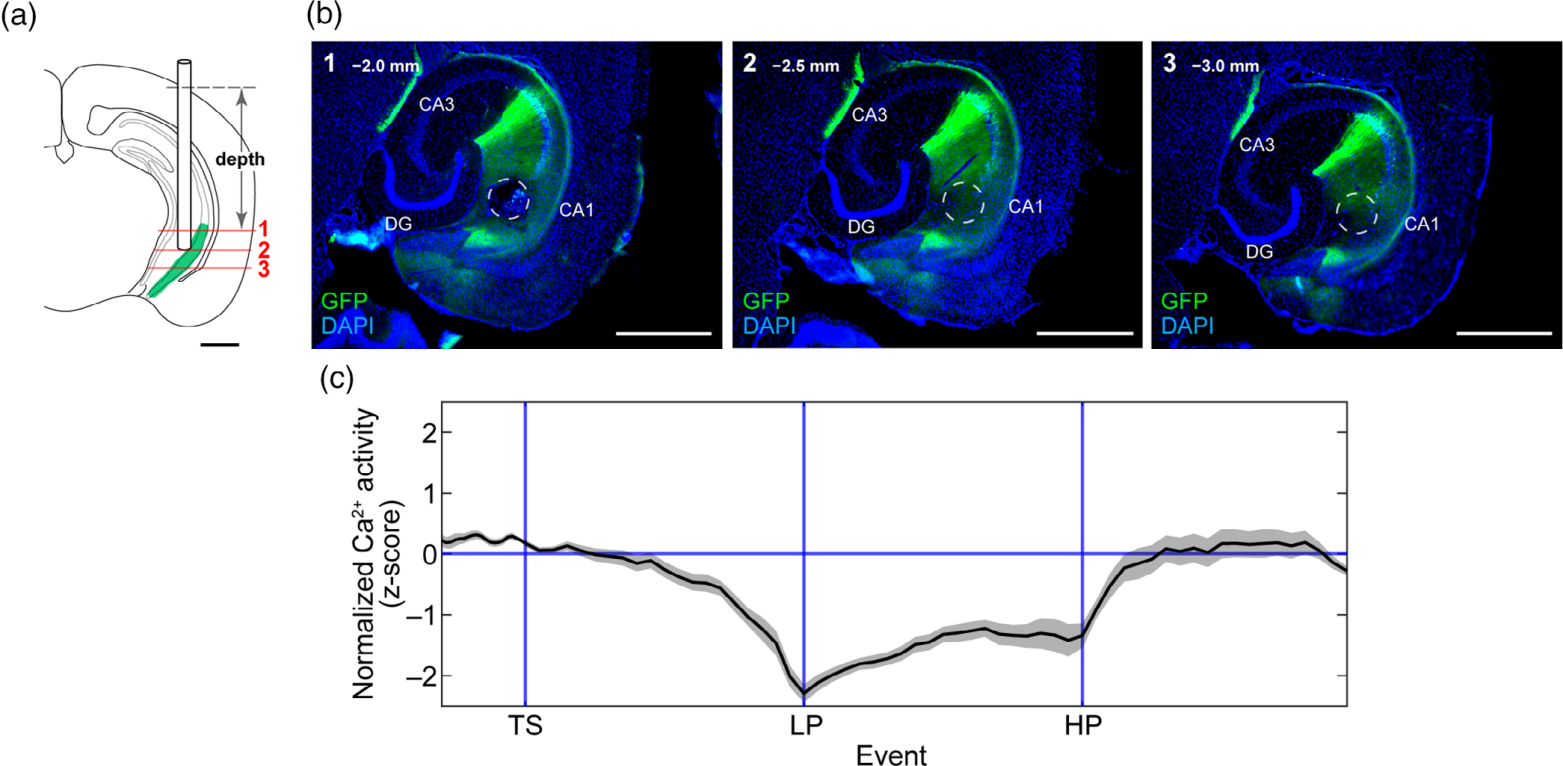
Assessment of compound Ca^2+^ activity of vCA1 neurons during an FR‐1 operant task. (a) Schematic illustration of the somal recordings from vCA1 neurons in Htr5B‐YC mice. (b) The images show YCnano50 expression (green) and optical fiber position. YCnano50 is expressed in hippocampal CA1 pyramidal neurons. In this image series, the order of the horizontal sections corresponds with the numbers in the schema (a), and the length represents the depth (mm) from the brain surface. The dashed circles indicate the location of the optical fiber. Fluorescence histology shows the expression of YCnano50 in the vCA1 (green) and the location of the optic fiber tip (white dashed circles). Scale bar, 1.0 mm. (c) Average trace of Ca^2+^ signals in the vCA1 in which the duration between trigger points was normalized (*n* = 9 mice); the shaded areas represent *SEM* [Color figure can be viewed at wileyonlinelibrary.com]

We found that vCA1^→LS^ neurons were markedly activated during the postprandial period. To understand if feeding‐related vCA1 activation is pathway specific, we investigated pan vCA1 neuronal activity during the FR‐1 task. We used Htr5B‐YC bitransgenic mice, in which YC was expressed in pan‐CA1 pyramidal neurons (Yoshida et al., 2019) (Figure 3a,b). We observed similar dynamic changes in pan‐vCA1 neurons in the TS‐HP phases; in particular, at the time of lever press, vCA1 activity was suppressed. We had previously demonstrated that the suppression of vCA1 activity at the time of lever press is necessary for goal‐directed action (Yoshida et al., 2019). After the HP phase, pan‐vCA1 activity did not exhibit a surge, which was distinct from vCA1^→LS^ activity (Figure 3c). These results demonstrate that feeding‐related vCA1 activation is vCA1^→LS^ pathway specific and suggest that there are vCA1 neurons that project to other brain regions and exhibit inhibition during the consummatory phase.

**FIGURE 4:**
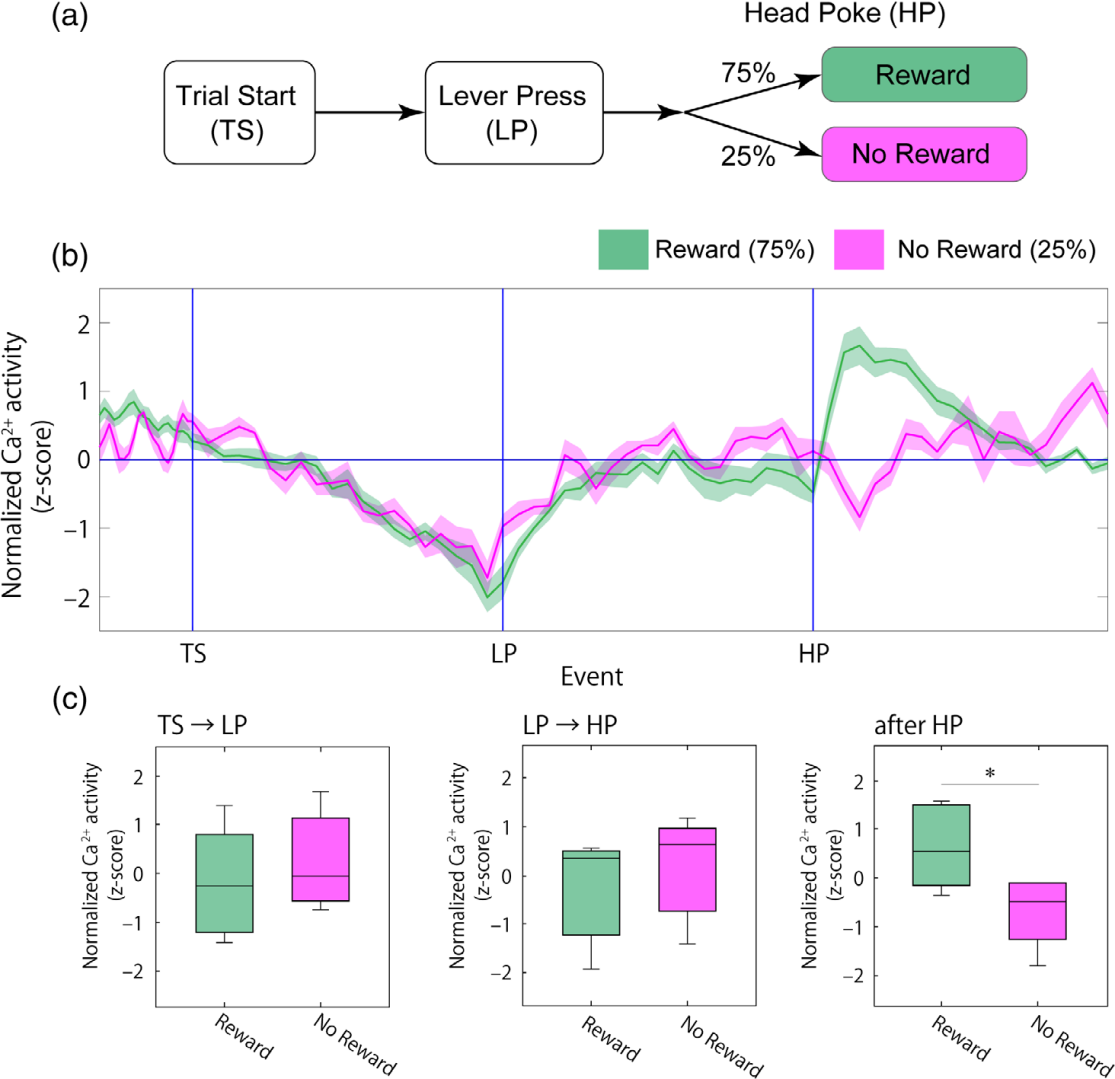
Assessment of compound Ca^2+^ activity in vCA1^→LS^ input neurons during a 75% rewarded variable ratio (VR) schedule operant task. (a) Schematic illustration of the VR schedule operant task. (b) Average trace of Ca^2+^ signals in the reward delivery (green) and reward omission trials (magenta; *n* = 9 mice). The reward was given randomly in 75% of the trials. The shaded areas represent *SEM*. (c) Averaged Ca^2+^ activity during the TS‐LP, LP‐HP, and ITI periods, respectively (two‐sided paired *t*‐test; TS → LP, *t*(8) = 1.71, *p* = .16; LP → HP, *t*(8) = 1.01, *p* = .37; after HP, *t*(8) = 2.82, *p* = .048). In the box plots, the horizontal lines within the boxes indicate the median, and the bottom and top edges of the boxes indicate the 25th and 75th percentiles, respectively; *p < .05 [Color figure can be viewed at wileyonlinelibrary.com]

We next sought to address if feeding‐related vCA1 activity is contingent on the presence of food. To evaluate this, we employed a variable ratio (VR) schedule. In this task, a reward pellet was given at 75% probability and was not given at 25% probability when mice pressed an active lever (Figure 4a). From the TS to HP phases, vCA1^→LS^ activity was indistinguishable between rewarded and non‐rewarded trials. However, after HP, the vCA1^→LS^ activity surge disappeared in the non‐rewarded trials (Figure 4b,c), indicating the contingency of vCA1^→LS^ activity on feeding.

**FIGURE 5:**
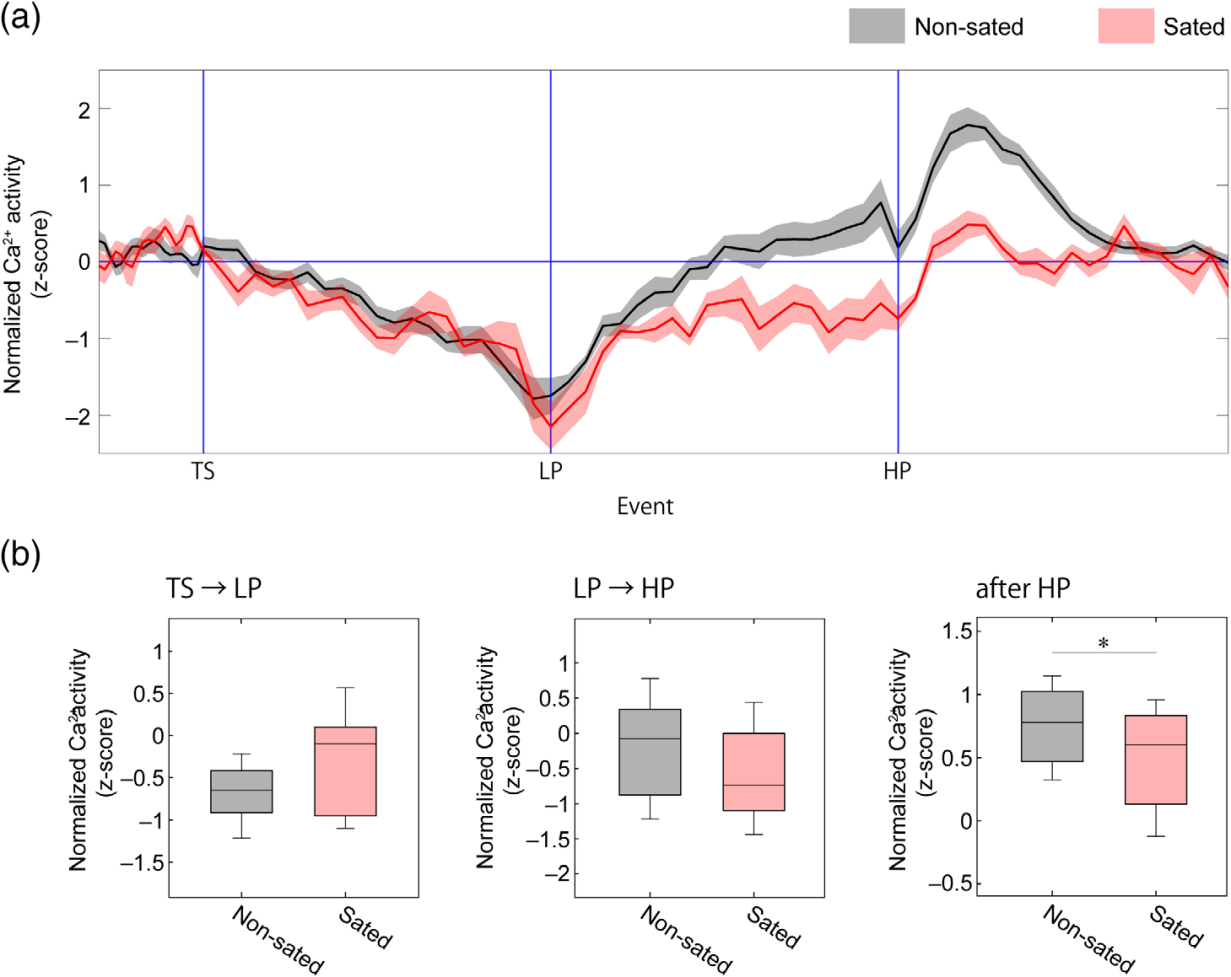
Assessment of compound Ca^2+^ activity in vCA1^→LS^ input neurons during an FR‐1 operant task under sated conditions. (a) Average trace of Ca^2+^ signals in vCA1^→LS^ input neurons under non‐sated (*n* = 12 mice) or sated conditions (*n* = 12 mice). Trace of Ca^2+^ signals in non‐sated conditions corresponding with Figure 2c. The shaded areas represent *SEM*. (b) Average Ca^2+^ activity during the TS‐LP, LP‐HP, and ITI periods, respectively (two‐sided paired *t*‐test; TS → LP, *t*(11) = 1.69, *p* = .13; LP → HP, *t*(11) = 0.62, *p* = .55; after HP, *t*(11) = 2.95, *p* = .018). In the box plots, the horizontal lines within the boxes indicate the median, and the bottom and top edges of the boxes indicate the 25th and 75th percentiles, respectively; *p < .05 [Color figure can be viewed at wileyonlinelibrary.com]

We further examined whether such feeding‐related vCA1 activity coincided with either bodily state changes (e.g., ameliorating a hunger state) or food‐intake‐accompanied actions and external sensory perceptions, since the previous reward omission experiment could not exclude the influence of actions (chewing and swallowing) and sensations (olfaction and taste). Mice were fed normal chow for 3 hr before the task that decreased their motivation for the palatable pellets. Sated mice still pressed the lever to obtain palatable pellets (20 ± 11 presses/session) and ate the pellets, but the number of lever presses was significantly lower than in food‐restricted mice (45 ± 11 presses/session, *p* < .001). We concluded that sated mice pressed the lever and ate pellets as a habitual response. In this comparison, we found significantly lower vCA1^→LS^ activity after the HP phase in sated mice, suggesting that the gastrointestinal interoceptive response rather than food‐intake motions and external sensations were correlated with vCA1^→LS^ activity (Figure 5).

### 3.3 Activity of LS projecting vCA1 neurons was increased in anxious/aversive situations

**FIGURE 6:**
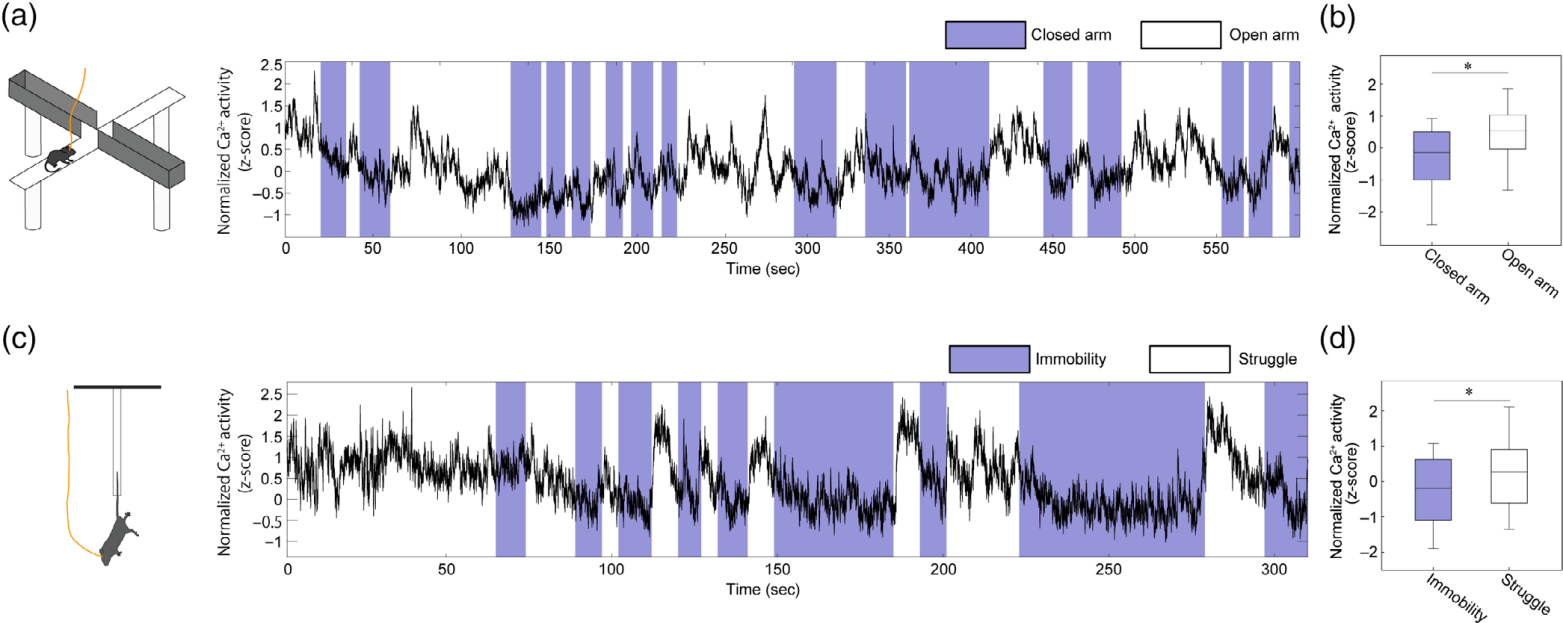
Assessment of compound Ca^2+^ activity in vCA1^→LS^ input neurons in the EPM and TST. (a) Representative Ca^2+^ activity of vCA1^→LS^ input neurons during the EPM. Blue shading indicates the periods when the mouse was in a closed arm. (b) The average Ca^2+^ amplitude in vCA1^→LS^ neurons was significantly higher in the open arms than in the closed arms (*n* = 10 mice, two‐sided paired *t*‐test, *t*(9) = 4.77, *p* = .0010). (c) Representative Ca^2+^ activity of vCA1^→LS^ input neurons during the TST. Blue shading indicates the periods when the mouse was in immobile. (d) The average Ca^2+^ amplitude in vCA1^→LS^ neurons was significantly higher during struggle than during immobility (*n* = 12 mice, two‐sided paired *t*‐test*, t*(11) = 3.27, *p* = .0075); *p < .01 [Color figure can be viewed at wileyonlinelibrary.com]

vCA1 neurons are known to engage in anxiety or avoidance behavior. Specifically, vCA1 neurons are activated in anxious situations or during an active coping behavior to avoid aversive situations (Jimenez et al., 2018). However, it was unclear if our selected population of vCA1^→LS^ neurons behaved similar to or unlike pan‐vCA1 neurons in these situations. To test this, we recorded vCA1^→LS^ activity in an elevated plus maze (EPM) and tail suspension test (TST). In the EPM, mice typically avoid open arms because they innately feel anxious in open spaces (Pellow et al., 1985; Walf & Frye, 2007), but they will spend time in open arms to explore (time in the open: 33%). In our experiments, the activity of vCA1^→LS^ neurons was increased when mice were in the open arms (Figure 6a,b), consistent with the conventional view of pan‐vCA1 activity (Yoshida et al., 2019).

The TST is a paradigm designed to examine active (struggling) or passive (immobility) coping behavior in an aversive situation (Commons, Cholanians, Babb, & Ehlinger, 2017). In the present study, active coping (struggling time: 161 s) predominated during the initial exposure to suspension but this was typically replaced over time with the appearance of passive coping (immobility time: 153 s). In the TST, vCA1^→LS^ neuronal activity during active coping behaviors was higher than during passive coping behaviors (Figure 6c,d), indicating the shared activity of vCA1^→LS^ neurons in aversive situations. These results demonstrate that vCA1^→LS^ neurons are activated in anxious or aversive situations, as widely accepted for pan‐vCA1 neurons, in spite of the specific vCA1^→LS^ neuronal activities during feeding.

## 4 DISCUSSION

Here we demonstrated that the vCA1^→LS^ neurons, but not pan‐vCA1 neurons, were activated during feeding. vCA1^→LS^ activity was correlated with gastrointestinal interoceptive responses rather than food intake‐related motions or exteroceptive responses. Thus, in addition to pan‐vCA1 neurons, this specific pathway is also activated in both aversive and anxious situations.

In previous studies, vHip lesions resulted in the enhancement of feeding in rats (Davidson et al., 2009; Davidson & Jarrard, 1993; Hock Jr. & Bunsey, 1998), indicating that the vHip controls feeding behavior. Results from artificially manipulating the vHip were consistent with those of the lesion studies; the optogenetic and/or pharmacogenetic inactivation of the vHip promoted feeding while activation of the vHip suppressed feeding (Sweeney & Yang, 2015). Our observational experiments found unique vCA1 activation during the postprandial period. We propose the coding of vCA1^→LS^ neuronal activity in line with the model described by Kanoski and Grill (Kanoski & Grill, 2017).

Hippocampal neurons integrate episodic meal‐related memories and food‐relevant learned associations. These mnemonic processes are influenced by both external and internal contexts. Among the external contexts, visuospatial sensory information is primarily communicated to dorsal Hip (dHip) neurons (Webster, Ungerleider, & Bachevalier, 1991) and olfactory and gustatory sensory information is primarily communicated to vHip neurons (Fanselow & Dong, 2010; Mathiasen, Hansen, & Witter, 2015). Internal contexts include gastrointestinal interoceptive information and vHip neurons are thought to communicate interoceptive information (Kanoski & Grill, 2017). In a recent report, vHip ghrelin signaling increased meal size by counteracting the efficacy of various gut‐derived satiation signals (Suarez, Liu, Cortella, Noble, & Kanoski, 2020). Therefore, vHip neurons process olfactory/gustatory external contexts and gastrointestinal internal contexts. In this regard, vCA1^→LS^ neurons are unlikely to mediate such external contexts (Figure 5), rather they mediate the internal contexts (Figures 4 and 5). However, we should note the experimental outcomes with saccharin (Hannapel et al., 2019) in which vHip postmeal inhibition increased future saccharin intake. This suggests that vHip neurons preferentially process sweet external contexts over postprandial interoceptive contexts when there are minimal postingestive consequences, that is, no change in blood glucose. Further studies will be required to address the vHip function in terms of the external or internal context processing.

What does vCA1^→LS^ neuronal activity encode during the postprandial period? Here, we hypothesize that vCA1^→LS^ neurons encode predictive bodily state. In the food‐seeking lever‐press task, if the lever press was goal‐directed, a motivation would drive the pressing of the lever and the subject would attain the goal (i.e., obtain food). In this case, the prediction that a hunger state will be satisfied matches the outcome and vCA1^→LS^ neurons are activated. In the reward omission trial, the prediction does not match the outcome and vCA1^→LS^ neurons are not activated. If the lever press was habitual, the mouse would not predict the obtainment of food and vCA1^→LS^ neurons would not be activated, although the mice could also smell and taste the food. In line with our hypothesis, we further speculate that mice with vHip lesions or inactivation are induced to feed (Davidson et al., 2009; Davidson & Jarrard, 1993; Hock Jr. & Bunsey, 1998; Sweeney & Yang, 2015) because these mice cannot process information regarding predictive bodily state and may have lost the negative feedback from feeding. In contrast, continuous vHip activation may provide continuous false signals in which the predictive bodily state is satisfied, leading to suppressed feeding.

To fully test our hypothesis, it will be necessary to conduct an intervention of vCA1^→LS^ neuron activity with temporal precision. The pioneering work using pathway specific (vCA3^→dorsal LS^) optogenetic and/or pharmacogenetic long‐term vHip manipulation highlighted the causal relationship between vHip activity and feeding for the first time (Sweeney & Yang, 2015). Optogenetic activation of vHip neurons targeting the post‐meal period (~ 5 min illumination) resulted in the suppression of future feeding (Hannapel et al., 2019). However, the timing and duration of these studies were not based on vHip activity patterns, thus future studies should focus on timing‐specific (postprandial period), short‐term (on a scale of seconds) optogenetic manipulation to evaluate our proposal. In addition to testing our hypothesis, optogenetic manipulation would enable us to address vCA1 activity under sated conditions. We assumed that postprandial vCA1 activity under sated conditions would not be significantly higher than that 5 s before the trial start time, but it is also possible that there could be an increase in vCA1 after HP even under sated conditions. Specifically, the peak height of vCA1 activity should encode the bodily state, otherwise the difference between vCA1 activity pre‐ and post‐HP may be rather important. To discriminate between these, timing‐specific optogenetic inhibition would be ideal. If the former scenario is correct, it is predicted that optogenetic inhibition would disturb activity in both hungry and sated conditions.

The identity of the inputs that shape the activity of vCA1^→LS^ neurons during the postprandial period is currently unknown. Gastric distension is known to increase Hip neuron firing and metabolism (Xu, Sun, Lu, Tang, & Chen, 2008), and vagus nerve electrical stimulation increases Hip metabolism (Min, Tuor, & Chelikani, 2011; Wang et al., 2006). These gastrointestinal signals, therefore, are likely to shape vCA1^→LS^ neuronal activity during the postprandial period. In addition, vHip neurons express food‐related hormone receptors such as glucagon‐like peptide 1 (GLP‐1; Merchenthaler, Lane, & Shughrue, 1999) and the infusion of these hormones into the vHip alters feeding behavior (Hsu, Hahn, Konanur, Lam, & Kanoski, 2015; Kanoski et al., 2011). Signaling through these receptors could thus shape vHip activity. Further efforts will be required to verify the contribution of interoceptive information to vCA1^→LS^ neuronal activity.

In this study, we targeted vCA1^→LS^ neurons using a retrograde labeling approach. We must note that the targeted vCA1^→LS^ neurons also send collaterals into other brain regions (Gergues et al., 2020). Based on recently published data (Gergues et al., 2020), we inferred that approximately 8% of vCA1^→LS^ neurons send collaterals into the nucleus accumbens (NAc), which is known to be involved in reward‐related behaviors. Therefore, the compound Ca^2+^ activity we present here mainly consists of that from vCA1^→NAc^ neurons. While it would be difficult to estimate the proportion of concomitant vCA1^→NAc^ activity based on anatomical information, we must recognize this potential limitation in the interpretation of our results.

Pharmacological activation of vCA1/CA3^→LS^ neurons has been shown to decrease anxiety, while activation of vCA1^→PFC^ neurons promotes anxiety (Parfitt et al., 2017). We found that vCA1^→LS^ neurons were activated in aversive or anxious situations. Collectively, the data suggest that vCA1^→LS^ neurons may alleviate anxiety levels in anxious situations. However, at present, it is currently difficult to reconcile the functions of vCA1^→LS^ neurons in feeding with those in anxiety. Behavioral tests utilizing conditions of anxiety and food intake, such as a novelty‐induced hypophagia test (Dulawa & Hen, 2005) or a novelty suppressed feeding test (Santarelli et al., 2003), may provide opportunities to address the biological significance of the multi‐functional vCA1^→LS^ neurons.

## CONFLICTS OF INTEREST

The authors declare no conflicts of interest.

## Data Availability

The data that support the findings of this study are available from the corresponding author upon reasonable request.

## References

[hipo23289-bib-0001] Anthony, T. E. , Dee, N. , Bernard, A. , Lerchner, W. , Heintz, N. , & Anderson, D. J. (2014). Control of stress‐induced persistent anxiety by an extra‐amygdala septohypothalamic circuit. Cell, 156(3), 522–536. 10.1016/j.cell.2013.12.040 24485458 PMC3982923

[hipo23289-bib-0002] Bagot, R. C. , Parise, E. M. , Peña, C. J. , Zhang, H. X. , Maze, I. , Chaudhury, D. , … Nestler, E. J. (2015). Ventral hippocampal afferents to the nucleus accumbens regulate susceptibility to depression. Nature Communications, 6, 7062. 10.1038/ncomms8062 PMC4430111 25952660

[hipo23289-bib-0003] Beatty, W. W. , & Schwartzbaum, J. S. (1968). Consummatory behavior for sucrose following septal lesions in the rat. Journal of Comparative and Physiological Psychology, 65(1), 93–102. 10.1037/h0025389 5648471

[hipo23289-bib-0004] Bienkowski, M. S. , Bowman, I. , Song, M. Y. , Gou, L. , Ard, T. , Cotter, K. , … Dong, H. W. (2018). Integration of gene expression and brain‐wide connectivity reveals the multiscale organization of mouse hippocampal networks. Nature Neuroscience, 21(11), 1628–1643. 10.1038/s41593-018-0241-y 30297807 PMC6398347

[hipo23289-bib-0005] Ciocchi, S. , Passecker, J. , Malagon‐Vina, H. , Mikus, N. , & Klausberger, T. (2015). Brain computation. Selective information routing by ventral hippocampal CA1 projection neurons. Science, 348(6234), 560–563. 10.1126/science.aaa3245 25931556

[hipo23289-bib-0006] Commons, K. G. , Cholanians, A. B. , Babb, J. A. , & Ehlinger, D. G. (2017). The rodent forced swim test measures stress‐coping strategy, not depression‐like behavior. ACS Chemical Neuroscience, 8(5), 955–960. 10.1021/acschemneuro.7b00042 28287253 PMC5518600

[hipo23289-bib-0007] Davidson, T. L. , Chan, K. , Jarrard, L. E. , Kanoski, S. E. , Clegg, D. J. , & Benoit, S. C. (2009). Contributions of the hippocampus and medial prefrontal cortex to energy and body weight regulation. Hippocampus, 19(3), 235–252. 10.1002/hipo.20499 18831000 PMC2649976

[hipo23289-bib-0008] Davidson, T. L. , & Jarrard, L. E. (1993). A role for hippocampus in the utilization of hunger signals. Behavioral and Neural Biology, 59(2), 167–171. 10.1016/0163-1047(93)90925-8 8476385

[hipo23289-bib-0009] Deng, K. , Yang, L. , Xie, J. , Tang, H. , Wu, G.‐S. , & Luo, H.‐R. (2019). Whole‐brain mapping of projection from mouse lateral septal nucleus. Biology Open, 8(7), bio043554. 10.1242/bio.043554 31208998 PMC6679409

[hipo23289-bib-0010] Dulawa, S. C. , & Hen, R. (2005). Recent advances in animal models of chronic antidepressant effects: The novelty‐induced hypophagia test. Neuroscience and Biobehavioral Reviews, 29(4–5), 771–783. 10.1016/j.neubiorev.2005.03.017 15890403

[hipo23289-bib-0011] Fanselow, M. S. , & Dong, H.‐W. (2010). Are the dorsal and ventral hippocampus functionally distinct structures? Neuron, 65(1), 7–19. 10.1016/j.neuron.2009.11.031 20152109 PMC2822727

[hipo23289-bib-0012] Gergues, M. M. , Han, K. J. , Choi, H. S. , Brown, B. , Clausing, K. J. , Turner, V. S. , … Kheirbek, M. A. (2020). Circuit and molecular architecture of a ventral hippocampal network. Nature Neuroscience, 23(11), 1444–1452. 10.1038/s41593-020-0705-8 32929245 PMC7606799

[hipo23289-bib-0013] Hannapel, R. , Ramesh, J. , Ross, A. , LaLumiere, R. T. , Roseberry, A. G. , & Parent, M. B. (2019). Postmeal optogenetic inhibition of dorsal or ventral hippocampal pyramidal neurons increases future intake. eNeuro, 6(1), 1–16. 10.1523/ENEURO.0457-18.2018 PMC6348449 30693314

[hipo23289-bib-0014] Hannapel, R. C. , Henderson, Y. H. , Nalloor, R. , Vazdarjanova, A. , & Parent, M. B. (2017). Ventral hippocampal neurons inhibit postprandial energy intake. Hippocampus, 27(3), 274–284. 10.1002/hipo.22692 28121049

[hipo23289-bib-0015] Hock, B. J., Jr. , & Bunsey, M. D. (1998). Differential effects of dorsal and ventral hippocampal lesions. The Journal of Neuroscience, 18(17), 7027–7032. 10.1523/JNEUROSCI.18-17-07027.1998 9712671 PMC6792982

[hipo23289-bib-0016] Hsu, T. M. , Hahn, J. D. , Konanur, V. R. , Lam, A. , & Kanoski, S. E. (2015). Hippocampal GLP‐1 receptors influence food intake, meal size, and effort‐based responding for food through volume transmission. Neuropsychopharmacology, 40(2), 327–337. 10.1038/npp.2014.175 25035078 PMC4443945

[hipo23289-bib-0017] Hsu, T. M. , Suarez, A. N. , & Kanoski, S. E. (2016). Ghrelin: A link between memory and ingestive behavior. Physiology & Behavior, 162, 10–17. 10.1016/j.physbeh.2016.03.039 27072509 PMC4899147

[hipo23289-bib-0018] Jimenez, J. C. , Su, K. , Goldberg, A. R. , Luna, V. M. , Biane, J. S. , Ordek, G. , … Kheirbek, M. A. (2018). Anxiety cells in a hippocampal‐hypothalamic circuit. Neuron, 97(3), 670–683.e676. 10.1016/j.neuron.2018.01.016 29397273 PMC5877404

[hipo23289-bib-0019] Kanemaru, K. , Sekiya, H. , Xu, M. , Satoh, K. , Kitajima, N. , Yoshida, K. , … Tanaka, K. F. (2014). In vivo visualization of subtle, transient, and local activity of astrocytes using an ultrasensitive Ca(2+) indicator. Cell Reports, 8(1), 311–318. 10.1016/j.celrep.2014.05.056 24981861

[hipo23289-bib-0020] Kanoski, S. E. , & Grill, H. J. (2017). Hippocampus contributions to food intake control: Mnemonic, neuroanatomical, and endocrine mechanisms. Biological Psychiatry, 81(9), 748–756. 10.1016/j.biopsych.2015.09.011 26555354 PMC4809793

[hipo23289-bib-0021] Kanoski, S. E. , Hayes, M. R. , Greenwald, H. S. , Fortin, S. M. , Gianessi, C. A. , Gilbert, J. R. , & Grill, H. J. (2011). Hippocampal leptin signaling reduces food intake and modulates food‐related memory processing. Neuropsychopharmacology, 36(9), 1859–1870. 10.1038/npp.2011.70 21544068 PMC3154104

[hipo23289-bib-0022] King, T. R. , & Nance, D. M. (1986). Neuroestrogenic control of feeding behavior and body weight in rats with kainic acid lesions of the lateral septal area. Physiology & Behavior, 37(3), 475–481. 10.1016/0031-9384(86)90209-x 3749306

[hipo23289-bib-0023] Kobayashi, K. , Sano, H. , Kato, S. , Kuroda, K. , Nakamuta, S. , Isa, T. , … Kobayashi, K. (2016). Survival of corticostriatal neurons by Rho/Rho‐kinase signaling pathway. Neuroscience Letters, 630, 45–52. 10.1016/j.neulet.2016.07.020 27424794

[hipo23289-bib-0024] Maniam, J. , & Morris, M. J. (2012). The link between stress and feeding behaviour. Neuropharmacology, 63(1), 97–110. 10.1016/j.neuropharm.2012.04.017 22710442

[hipo23289-bib-0025] Mathiasen, M. L. , Hansen, L. , & Witter, M. P. (2015). Insular projections to the parahippocampal region in the rat. The Journal of Comparative Neurology, 523(9), 1379–1398. 10.1002/cne.23742 25641117

[hipo23289-bib-0026] Merchenthaler, I. , Lane, M. , & Shughrue, P. (1999). Distribution of pre‐pro‐glucagon and glucagon‐like peptide‐1 receptor messenger RNAs in the rat central nervous system. The Journal of Comparative Neurology, 403(2), 261–280. 10.1002/(sici)1096-9861(19990111)403:2<261::aid-cne8>3.0.co;2-5 9886047

[hipo23289-bib-0027] Min, D. K. , Tuor, U. I. , & Chelikani, P. K. (2011). Gastric distention induced functional magnetic resonance signal changes in the rodent brain. Neuroscience, 179, 151–158. 10.1016/j.neuroscience.2011.01.051 21284950

[hipo23289-bib-0028] Natsubori, A. , Tsutsui‐Kimura, I. , Nishida, H. , Bouchekioua, Y. , Sekiya, H. , Uchigashima, M. , … Tanaka, K. F. (2017). Ventrolateral striatal medium spiny neurons positively regulate food‐incentive, goal‐directed behavior independently of D1 and D2 selectivity. The Journal of Neuroscience, 37(10), 2723–2733. 10.1523/JNEUROSCI.3377-16.2017 28167674 PMC6596637

[hipo23289-bib-0029] Padilla‐Coreano, N. , Bolkan, S. S. , Pierce, G. M. , Blackman, D. R. , Hardin, W. D. , Garcia‐Garcia, A. L. , … Gordon, J. A. (2016). Direct ventral hippocampal‐prefrontal input is required for anxiety‐related neural activity and behavior. Neuron, 89(4), 857–866. 10.1016/j.neuron.2016.01.011 26853301 PMC4760847

[hipo23289-bib-0030] Parfitt, G. M. , Nguyen, R. , Bang, J. Y. , Aqrabawi, A. J. , Tran, M. M. , Seo, D. K. , … Kim, J. C. (2017). Bidirectional control of anxiety‐related behaviors in mice: Role of inputs arising from the ventral hippocampus to the lateral septum and medial prefrontal cortex. Neuropsychopharmacology, 42(8), 1715–1728. 10.1038/npp.2017.56 28294135 PMC5518909

[hipo23289-bib-0031] Pellow, S. , Chopin, P. , File, S. E. , & Briley, M. (1985). Validation of open:Closed arm entries in an elevated plus‐maze as a measure of anxiety in the rat. Journal of Neuroscience Methods, 14(3), 149–167. 10.1016/0165-0270(85)90031-7 2864480

[hipo23289-bib-0032] Pentkowski, N. S. , Litvin, Y. , Blanchard, D. C. , Vasconcellos, A. , King, L. B. , & Blanchard, R. J. (2009). Effects of acidic‐astressin and ovine‐CRF microinfusions into the ventral hippocampus on defensive behaviors in rats. Hormones and Behavior, 56(1), 35–43. 10.1016/j.yhbeh.2009.02.007 19269291 PMC2773020

[hipo23289-bib-0033] Santarelli, L. , Saxe, M. , Gross, C. , Surget, A. , Battaglia, F. , Dulawa, S. , … Hen, R. (2003). Requirement of hippocampal neurogenesis for the behavioral effects of antidepressants. Science, 301(5634), 805–809. 10.1126/science.1083328 12907793

[hipo23289-bib-0034] Schumacher, A. , Villaruel, F. R. , Ussling, A. , Riaz, S. , Lee, A. C. H. , & Ito, R. (2018). Ventral hippocampal CA1 and CA3 differentially mediate learned approach‐avoidance conflict processing. Current Biology, 28(8), 1318–1324.e1314. 10.1016/j.cub.2018.03.012 29606418

[hipo23289-bib-0035] Singewald, G. M. , Rjabokon, A. , Singewald, N. , & Ebner, K. (2011). The modulatory role of the lateral septum on neuroendocrine and behavioral stress responses. Neuropsychopharmacology, 36(4), 793–804. 10.1038/npp.2010.213 21160468 PMC3055728

[hipo23289-bib-0036] Suarez, A. N. , Liu, C. M. , Cortella, A. M. , Noble, E. E. , & Kanoski, S. E. (2020). Ghrelin and orexin interact to increase meal size through a descending hippocampus to hindbrain signaling pathway. Biological Psychiatry, 87(11), 1001–1011. 10.1016/j.biopsych.2019.10.012 31836175 PMC7188579

[hipo23289-bib-0037] Sweeney, P. , & Yang, Y. (2015). An excitatory ventral hippocampus to lateral septum circuit that suppresses feeding. Nature Communications, 6, 10188–10188. 10.1038/ncomms10188 PMC4682174 26666960

[hipo23289-bib-0038] Tanaka, K. F. , & Hamaguchi, T. (2019). Translational approach to apathy‐like behavior in mice: From the practical point of view. Psychiatry and Clinical Neurosciences, 73(11), 685–689. 10.1111/pcn.12915 31304614

[hipo23289-bib-0039] Tanaka, K. F. , Matsui, K. , Sasaki, T. , Sano, H. , Sugio, S. , Fan, K. , … Yamanaka, A. (2012). Expanding the repertoire of optogenetically targeted cells with an enhanced gene expression system. Cell Reports, 2(2), 397–406. 10.1016/j.celrep.2012.06.011 22854021

[hipo23289-bib-0040] Tervo, D. G. , Hwang, B. Y. , Viswanathan, S. , Gaj, T. , Lavzin, M. , Ritola, K. D. , … Karpova, A. Y. (2016). A designer AAV variant permits efficient retrograde access to projection neurons. Neuron, 92(2), 372–382. 10.1016/j.neuron.2016.09.021 27720486 PMC5872824

[hipo23289-bib-0041] Ulrich‐Lai, Y. M. , & Ryan, K. K. (2014). Neuroendocrine circuits governing energy balance and stress regulation: Functional overlap and therapeutic implications. Cell Metabolism, 19(6), 910–925. 10.1016/j.cmet.2014.01.020 24630812 PMC4047143

[hipo23289-bib-0042] Walf, A. A. , & Frye, C. A. (2007). The use of the elevated plus maze as an assay of anxiety‐related behavior in rodents. Nature Protocols, 2(2), 322–328. 10.1038/nprot.2007.44 17406592 PMC3623971

[hipo23289-bib-0043] Wang, G.‐J. , Yang, J. , Volkow, N. D. , Telang, F. , Ma, Y. , Zhu, W. , … Fowler, J. S. (2006). Gastric stimulation in obese subjects activates the hippocampus and other regions involved in brain reward circuitry. Proceedings of the National Academy of Sciences of the United States of America, 103(42), 15641–15645. 10.1073/pnas.0601977103 17023542 PMC1592230

[hipo23289-bib-0044] Webster, M. J. , Ungerleider, L. G. , & Bachevalier, J. (1991). Connections of inferior temporal areas TE and TEO with medial temporal‐lobe structures in infant and adult monkeys. The Journal of Neuroscience, 11(4), 1095–1116. 10.1523/JNEUROSCI.11-04-01095.1991 2010806 PMC6575389

[hipo23289-bib-0045] Xu, L. , Sun, X. , Lu, J. , Tang, M. , & Chen, J. D. (2008). Effects of gastric electric stimulation on gastric distention responsive neurons and expressions of CCK in rodent hippocampus. Obesity, 16(5), 951–957. 10.1038/oby.2008.17 18309302

[hipo23289-bib-0046] Yoshida, K. , Drew, M. R. , Mimura, M. , & Tanaka, K. F. (2019). Serotonin‐mediated inhibition of ventral hippocampus is required for sustained goal‐directed behavior. Nature Neuroscience, 22(5), 770–777. 10.1038/s41593-019-0376-5 30988523

